# Comparative Analysis of the Codon Usage Pattern in the Chloroplast Genomes of Gnetales Species

**DOI:** 10.3390/ijms251910622

**Published:** 2024-10-02

**Authors:** Xiaoming Yang, Yuan Wang, Wenxuan Gong, Yinxiang Li

**Affiliations:** 1 Co-Innovation Center for Sustainable Forestry in Southern China, Nanjing Forestry University, Nanjing 210037, China; xmyang@njfu.edu.cn; 2 Inner Mongolia Academy of Forestry Science, Hohhot 010021, China; yznx41.5@163.com (W.G.); 13015209918@163.com (Y.L.)

**Keywords:** codon usage pattern, chloroplast genome, phylogenetic analysis, Gnetales

## Abstract

Codon usage bias refers to the preferential use of synonymous codons, a widespread phenomenon found in bacteria, plants, and animals. Codon bias varies among species, families, and groups within kingdoms and between genes within an organism. Codon usage bias (CUB) analysis sheds light on the evolutionary dynamics of various species and optimizes targeted gene expression in heterologous host plants. As a significant order of gymnosperms, species within Gnetales possess extremely high ecological and pharmaceutical values. However, comprehensive analyses of CUB within the chloroplast genomes of Gnetales species remain unexplored. A systematic analysis was conducted to elucidate the codon usage patterns in 13 diverse Gnetales species based on the chloroplast genomes. Our results revealed that chloroplast coding sequences (cp CDSs) in 13 Gnetales species display a marked preference for AT bases and A/T-ending codons. A total of 20 predominantly high-frequency codons and between 2 and 7 optimal codons were identified across these species. The findings from the ENC-plot, PR2-plot, and neutrality analyses suggested that both mutation pressure and natural selection exert influence on the codon bias in these 13 Gnetales species, with natural selection emerging as the predominant influence. Correspondence analysis (COA) demonstrated variation in the codon usage patterns among the Gnetales species and indicated mutation pressure is another factor that could impact CUB. Additionally, our research identified a positive correlation between the measure of idiosyncratic codon usage level of conservatism (MILC) and synonymous codon usage order (SCUO) values, indicative of CUB’s potential influence on gene expression. The comparative analysis concerning codon usage frequencies among the 13 Gnetales species and 4 model organisms revealed that *Saccharomyces cerevisiae* and *Nicotiana tabacum* were the optimal exogenous expression hosts. Furthermore, the cluster and phylogenetic analyses illustrated distinct patterns of differentiation, implying that codons, even with weak or neutral preferences, could affect the evolutionary trajectories of these species. Our results reveal the characteristics of codon usage patterns and contribute to an enhanced comprehension of evolutionary mechanisms in Gnetales species.

## 1. Introduction

The chloroplast, the primary organelle in green plants responsible for metabolic and photosynthetic processes, provides essential energy for plant growth and development [1,2]. Compared with the nuclear genome, genetic information is more conserved in the chloroplast genome. Generally, the chloroplast harbors a conserved genomic structure that is small, self-replicating, and demonstrates single-parental or bi-parental inheritance along with a stable evolutionary rate and a low nucleotide substitution rate [2]. The majority of chloroplast genomes consist of double-stranded closed circular DNA molecules, while some plants have a linear chloroplast genome structure [2]. Therefore, investigating the chloroplast genome holds significant importance for revealing evolutionary patterns, phylogenetic relationships, and species identification, and for understanding chloroplast gene expression and transformation among different plants [3,4].

Codons are essential to the transmission of genetic information, acting as a bridge between proteins, amino acids, and genetic materials within organisms. Codon degeneracy is defined as the phenomenon where most amino acids are represented by two to six associated codons [5]. Methionine (Met, encoded by AUG) and tryptophan (Trp, encoded by UGG) represent the only two amino acids with unique codons. Codon usage bias (CUB) characterizes the phenomenon of differential codon usage across various genes or genomes [6,7]. In many organisms, codon bias emerges in their genomes due to a combination of factors, including natural selection, mutational pressure, or random genetic drift [8,9]. Previous studies suggest that not only natural selection but also mutation pressure contribute to the codon usage bias in the chloroplast genomes of some species [9,10]. Furthermore, factors including GC content, gene length, gene expression level, and the evolutionary relationships of specific species, along with other genomic attributes, can affect the codon usage bias [11,12,13]. Exploring the characteristics of CUB in certain genes or genomes is crucial not only for understanding the molecular mechanisms of gene expression but also for aiding in the design of gene expression vectors to enhance the expression of the target gene [14]. Optimal codons can be employed to elucidate the molecular mechanisms by which organisms adapt to the external environment, thereby revealing the primary driving forces behind long-term molecular evolution in genomes [15].

Gnetales, an important order of gymnosperms, comprises three extant families (Ephedraceae, Gnetaceae, and Welwitschiaceae) and three extant genera (*Ephedra*, *Gnetum*, and *Welwitschia*). These genera and families demonstrate significant morphological and genetic differences compared with the remaining gymnosperm plants, such as Cycads, Ginkgo, and Coniferales. Furthermore, the precise classification of these plants remains a subject of debate [16,17]. Based on morphological and anatomical evidence, it has been proposed that Gnetales constitutes a sister group to angiosperms [17,18,19]. Additionally, *Gnetum* has exhibited a relatively low photosynthetic capacity, considered an inherent trait influenced by its unique evolutionary history [20]. In addition to their significant evolutionary divergence, certain species within the Gnetales group are valued for their medicinal, grain, and oil-producing qualities in various countries [21]. Leaves of Gnetales are rich in bioactive compounds, with significant functional and nutraceutical properties, such as stilbenes and flavonoids. These compounds are known for their significant anticarcinogenic and anti-inflammatory effects [22]. Furthermore, *G. africanum* and *G. buchholzianum* are grown as vegetables or cash crops in Africa, providing nutritional supplements that yield considerable economic benefits [23].

Recent analyses have decoded many chloroplast genomes and scrutinized their codon usage characteristics [6,7,8,9]. Even though the chloroplast genome sequences in Gnetales were available, the codon bias within these genomes was still not thoroughly investigated. In this study, we conducted a comprehensive analysis of codon usage patterns and their determinants. Moreover, we pinpointed the optimal strategies for enhancing the expression efficacy of foreign genes through chloroplasts and established phylogenetic connections within this group. Hence, examining codon usage patterns was vital for enriching our understanding of Gnetales species’ evolutionary progress and adaptability.

## 2. Results

### 2.1. Characteristics of Codon Usage Composition

**Figure 1:**
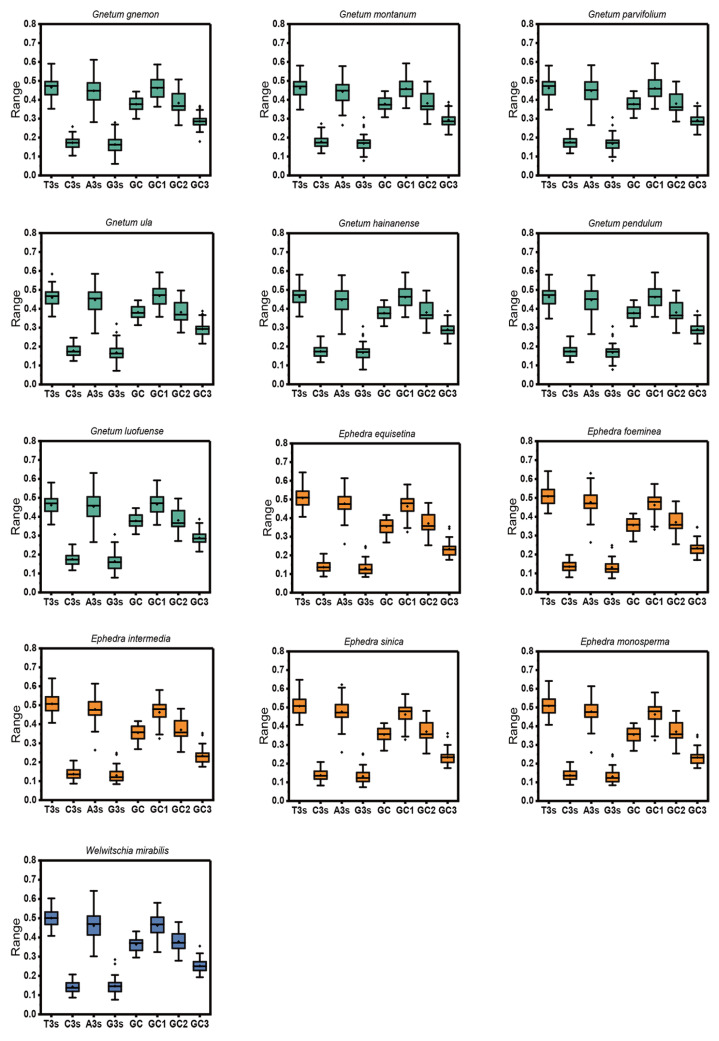
Distribution of nucleotide, overall GC content, GC1, GC2, and GC3 of cp CDSs in 13 Gnetales species.

After applying stringent filtering criteria, the count of identified cp CDSs ranged from 37 to 41 across 13 Gnetales species (Table S1). The nucleotide composition of cp CDSs from 13 species was analyzed, considering both the overall composition and the third codon position, to assess the influence of compositional properties (Figure 1). Results indicated that the mean T% content was the highest, followed by A%, C%, and G%, highlighting an unequal distribution of the four nucleotides in the chloroplast genome, with a preference for T-ending codons and then A-ending codons. The mean GC content and its components (GC1, GC2, and GC3) exhibited a range of values: 35.54% to 38.10%, 45.82% to 46.83%, 37.07% to 38.26%, and 23.34% to 39.35%, respectively. The GC content at all three codon positions, as well as the average across these positions, remained below 0.5, emphasizing a preference in the chloroplast genomes of 13 species for A/T bases and A/T-ending codons. Moreover, the GC content of the cp CDSs (GC1 > GC2 > GC3) indicated an uneven distribution across the three codon positions in 13 Gnetales species.

### 2.2. High-Frequency and Optimal Codons

**Figure 2:**
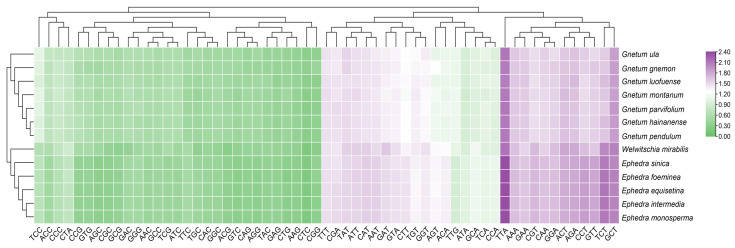
The RSCU values of cp CDSs across 13 Gnetales species are visualized, with a color gradient ranging from green to pink denoting an ascending average RSCU value for the codons.

Based on the relative synonymous codon usage (RSCU) values, we identified 20 codons with a prevalence (RSCU > 1) terminating in A/T nucleotides (7 in A and 13 in T), highlighting a marked preference for A/T-ending codons over G/C in 13 Gnetales species. Notably, TTA (encoding Leu) was the most favored codon (RSCU > 2.0), with GCT (encoding Ala) being a close second. Furthermore, RSCU values for identical codons across the species demonstrated significant uniformity (Figure 2).

Analyses employing the RFSC metrics also corroborated the same preference for A/T-ending codons (Table S2). A total of 11 HF codons were prevalent in all chloroplast genomes (TTA, GTT, TCT, ACT, TAT, CAA, AAA, GCT, GAA, AGA, GGA) in 13 Gnetales species. *Ephedra* species (*E. monosperma*, *E. equisetina*, *E. sinica*, and *W. mirabilis*) showed the highest prevalence of HF codons, with 18 each, while *Gnetum* species (*G. ula* and *G. luofuense*) had the lowest, with only 12.

The optimal codons for the 13 Gnetales species were identified utilizing the values of RSCU and ΔRSCU. Two and seven codons were affirmed as optimal for each species, yet no common optimal codons were established across the 13 Gnetales species (Table 1).

### 2.3. Neutrality Plot Analysis

**Figure 3:**
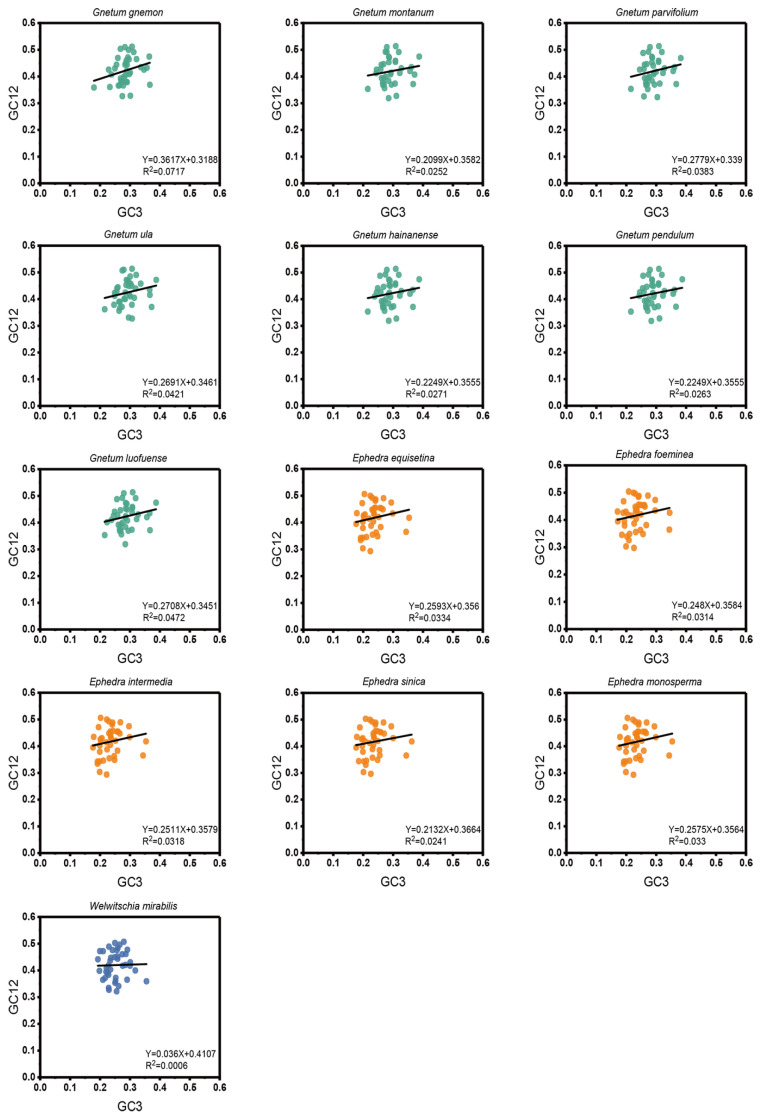
Neutrality plot of cp CDSs in different species to explore the relationship between GC12 and GC3. The black line represents the correlation line. The equation of the correlation line is shown at the bottom of the plot.

According to the neutrality plots (regression of GC12 on GC3) for the cp CDSs of the 13 Gnetales species, the distribution ranges of GC12 and GC3 were comparatively constrained, and most points were tightly clustered (Figure 3). The value of GC12 varied from 0.29 to 0.51, and GC3 varied from 0.17 to 0.39. There was an absence of a significant positive correlation between GC12 and GC3. Meanwhile, the slope of the regression line varied from 0.036 to 0.3617, suggesting that none of the species were subject to direct mutation pressure on the codon usage of the cp CDSs. Consequently, natural selection was likely to play a more significant role.

### 2.4. ENC-Plot Analysis

**Figure 4:**
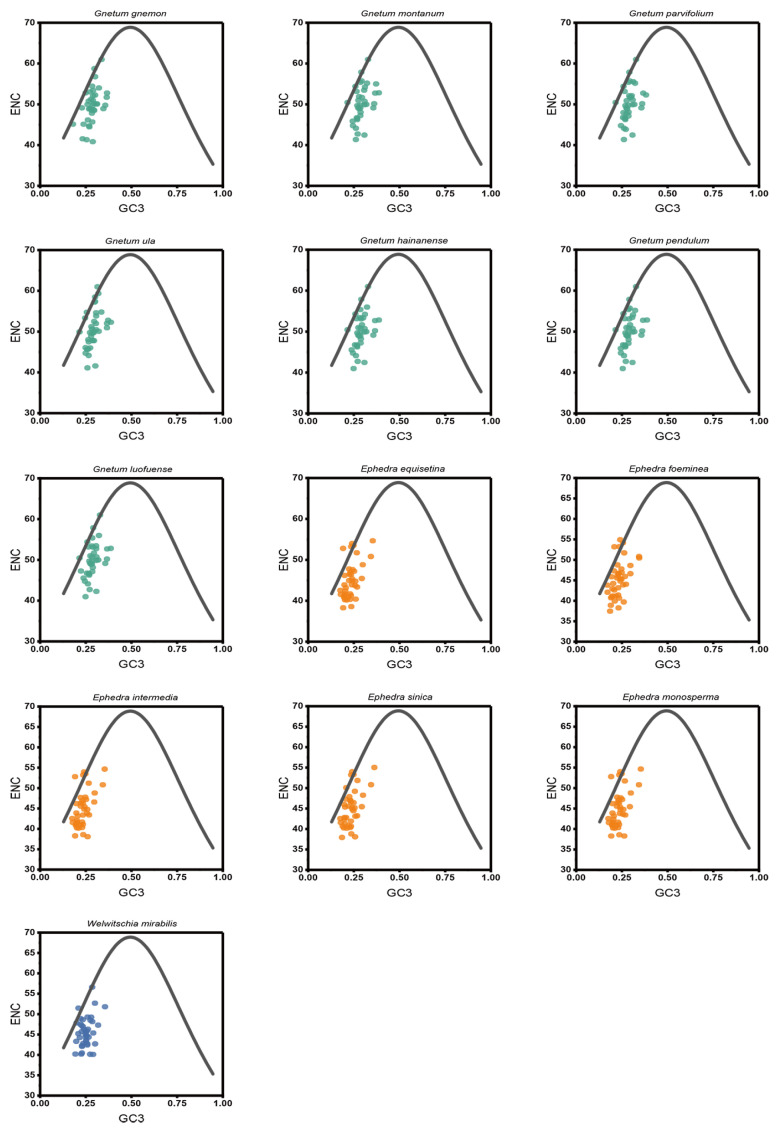
ENC-plot analysis of cp CDSs in 13 Gnetales species. If the point is distant from the standard curve, this suggests that the CUB of cp CDSs was primarily influenced by natural selection.

In the ENC-plot, a consistent distribution pattern of the effective number of codons (ENC) and GC3s was noted across the 13 Gnetales species, forming compact clusters on the left side (Figure 4). Most cp CDSs were positioned far from the standard curve, with only a few approaching it, indicative of the CUB of the cp CDSs being mainly influenced by natural selection. We conducted additional analysis on the ENC frequency distribution of cp CDSs in the 13 Gnetales species, emphasizing the disparities. The ENC ratio extended from −0.20 to 0.25. Among these, 22 to 28 cp CDSs, representing 53.70% to 71.80%, were situated within the range of −0.05 to 0.10 (Table S3).

### 2.5. PR2-Plot Analysis

**Figure 5:**
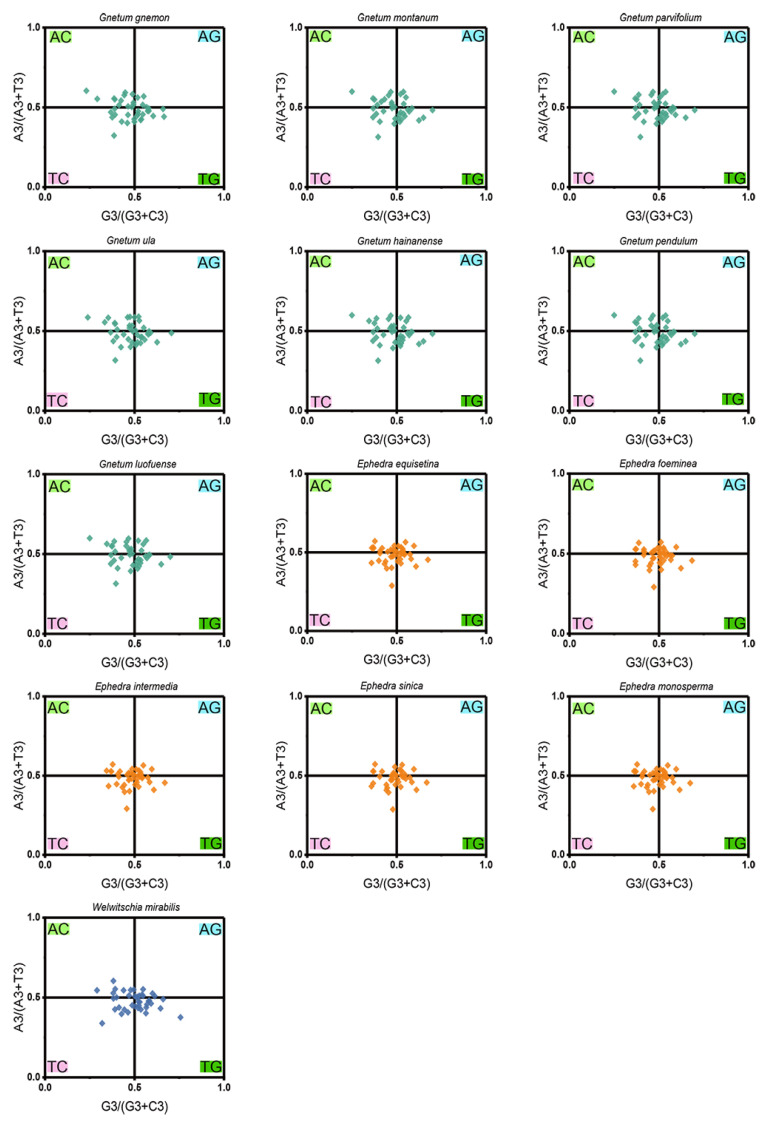
PR2-plot analysis of cp CDSs in 13 Gnetales species. GC bias and AT bias are on the abscissa axis and vertical axis, respectively.

PR2-plot analysis revealed a pronounced bias in nucleotide distribution, with a majority of the data points clustered in the bottom right quadrant (G3/(G3 + C3) > 0.5 and A3/(A3 + T3) > 0), which denotes a significant deviation in the use of the 4 nucleobases across the 13 Gnetales species (Figure 5). Specifically, the AT-bias values for all cp CDSs were consistently lower than 0.604, while the GC-bias values exceeded 0.230, highlighting a marked preference for T over A, and G over C, at the third codon position. This distinct A/T dominance in codon usage, coupled with the observed nucleotide distribution imbalance, suggests that both mutation pressure and natural selection were crucial in shaping codon usage patterns within the chloroplast genomes of the 13 Gnetales species.

### 2.6. Correspondence Analysis (COA)

**Figure 6:**
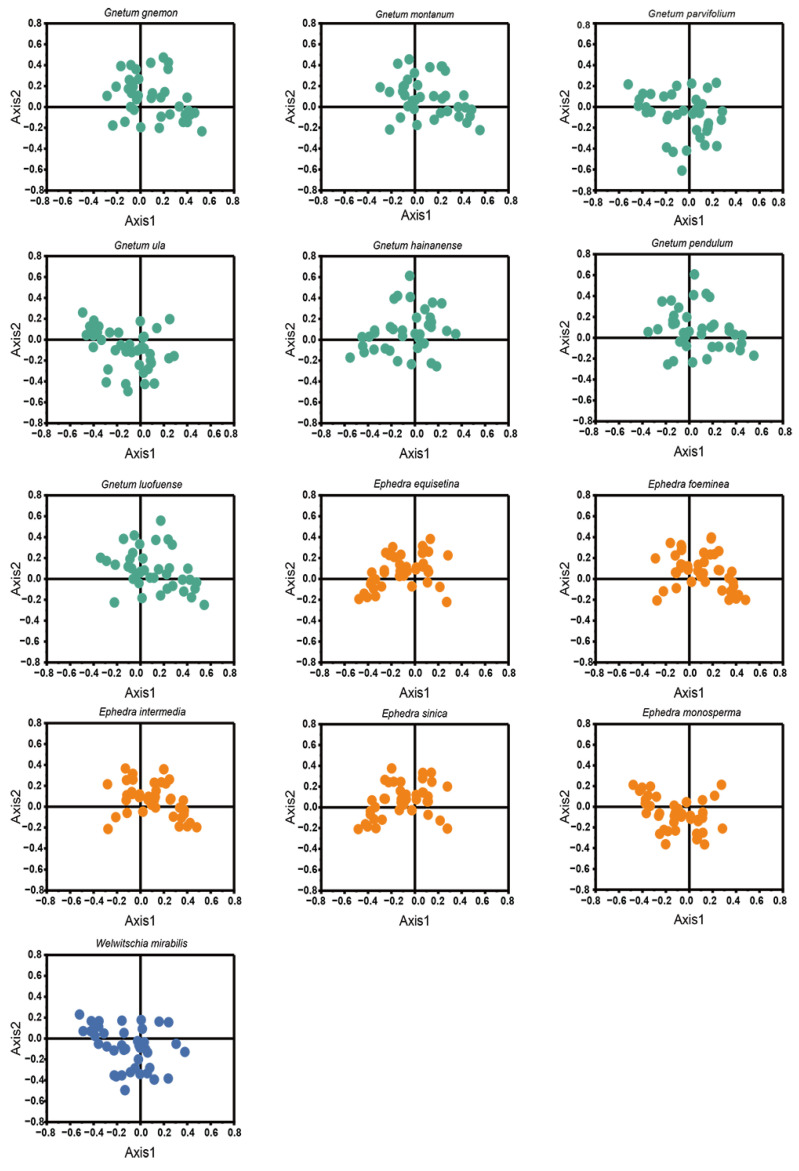
Correspondence analysis of cp CDSs in 13 Gnetales species.

Codon usage variation in 13 Gnetales species was examined via COA with cp CDSs. A comparison of the RSCU values of 59 codons revealed trends in codon usage among the Gnetales species, illustrated by the orthogonal axes (Figure 6). Points representing AT- and GC-ending codons were distinctly outlined, indicating divergent codon usage within Gnetales species. The first four axes explain an average of 50.74% of the total variation, with the first axis alone accounting for 25.63%, emphasizing the multifactorial influences on codon usage. Codons near the axes suggest that mutation pressure affected the CUB of cp CDSs.

Furthermore, correlation analysis incorporating CAI, CBI, Fop, GC3, GC, and L_aa on axis 1 identified factors affecting gene dispersion along axes 1 and 2. Results demonstrated a significant correlation between axis 1 and GC for all Gnetales species (*p* < 0.01), suggesting a strong influence of base composition, driven by mutation pressure, on codon usage preference (Table S4).

### 2.7. Relationship between CUB and Nucleotide Skews

The relationship between synonymous codon usage order (SCUO)and six nucleotide skewness was explored to evaluate the effects of skewness on CUB (Table S5). SCUO exhibited positive correlations with AT skew, purine skew, and keto skew across the 13 Gnetales species, while it was found to have negative correlations with GC skew, pyrimidine skew, and amino skew. Certain correlations were found to be significant (*p* < 0.01 or *p* < 0.05), indicating potential skewness influences on CUB. Furthermore, a significant positive correlation between SCUO and all six nucleotide skews was observed in both *G. gnemon* and *G. luofuense*. Therefore, we speculate that specific nucleotide skews significantly influenced the codon usage patterns of cp CDSs across the 13 Gnetales species.

### 2.8. Relationship between CUB and Protein Properties

The analysis of the relationship between CUB and protein properties was conducted across the 13 Gnetales species (Table S6). The grand average of hydropathicity (GRAVY) scores and gene lengths were found to have highly significant negative correlations with the SCUO values (*p* < 0.05), highlighting their substantial impact on the CUB of cp CDSs. Notably, *G. ula* demonstrated the strongest negative correlation (−0.617, *p* < 0.05) between SCUO and protein length, indicating elevated CUB in cp CDSs encoding smaller proteins. Our results suggest that shorter genes exhibit higher CUB, implying an inverse relationship between gene length and codon usage bias. Except for *E. sinica*, a low correlation between SCUO and aromaticity was observed in all species. *G. montanum* and *W. mirabilis* presented the most- and least-pronounced positive correlations between SCUO and the isoelectric point (PI), respectively.

### 2.9. Relationship between CUB and Gene Expression

High measure of idiosyncratic codon usage level of conservatism (MILC) values for each cp CDS of the 13 Gnetales species were found, indicating a high expression level of most cp CDSs (Table 2). Moreover, the relationship between SCUO and MILC elucidated the influence of CUB on gene expression dynamics (Table 3), with a pronounced positive correlation (*p* < 0.05 or *p* < 0.01) underscoring the significance of CUB in regulating gene expression. This significant association highlights the pivotal role of codon preferences in the modulation of gene expression levels, thereby offering insights into the potential gene regulation mechanism within the chloroplast genomes of the 13 Gnetales species.

### 2.10. Codon Usage Frequency among Different Species

Codon usage patterns of cp CDSs from the 13 Gnetales species were analyzed and compared with 4 model species. Results revealed minimal divergence in codon usage frequencies between the 13 Gnetales species and *A. thaliana*, *N. tabacum*, and *S. cerevisiae*, with differences in 12 to 17 (18.75–26.56%), 8 to 16 (12.50–25.00%), and 5 to 16 (7.81–25.00%) codons, respectively (Table S7). In contrast, a comparison with *E. coli* revealed a higher divergence, featuring 32 different codons. Therefore, *S. cerevisiae* and *N. tabacum* emerged as optimal heterologous expression hosts for Gnetales species.

### 2.11. Phylogenetic Analysis of Different Gnetales Species

**Figure 7:**
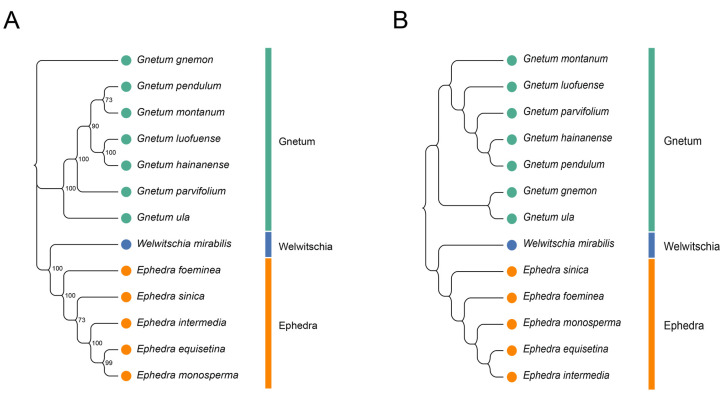
Phylogenetic and cluster analysis of 13 Gnetales species. (**A**) Phylogenetic analysis of chloroplast CDSs in the 13 Gnetales species. (**B**) Cluster analysis of RSCU values based on cp CDSs in the 13 Gnetales species. Different colors represent the family of different species, and the number on the node of each branch is the bootstrap value.

To assess the relationships among the 13 Gnetales species, we constructed a phylogenetic tree based on the cp CDSs (Figure 7A). All species were divided into three distinct clusters, and congeneric species were primarily grouped together. The findings indicate that high bootstrap values support most branches of the phylogenetic tree, demonstrating strong support for the identified groupings. The cluster tree, derived from the RSCU values of cp CDSs, showed a topology consistent with the phylogenetic tree (Figure 7B). However, some discrepancies remained between the two analyses, especially concerning the associations within the same genus. For instance, the phylogenetic tree indicated close relationships between *G. pendulum* and *G. montanum* and between *G. luofuense* and *G. hainanense*, while the clustering analysis associated *G. pendulum* more closely with *G. hainanense* and showed *G. luofuense* as independent. A similar pattern emerged in Ephedra; *E. equisetina* grouped with *E. monosperma* in the phylogenetic tree, but it was more closely related to *E. intermedia* in the RSCU-based tree.

## 3. Discussion

Codon preference denotes the differential selection of codons in genetic coding; these are crucial for the synthesis of proteins by specifying amino acids, thereby impacting gene regulation and evolution at the molecular level [6,8]. CUB is shaped by numerous biological factors including genomic architecture, gene expression levels, gene size, GC content, and the positional and neighboring-nucleotide context of codons, along with rates of recombination and mutation [6,9,24]. Enhanced scrutiny of chloroplast genomes has prompted investigations into codon preferences across various taxa; prominent examples include studies on Theaceae species [25], *Oryza* species [26], and *Gynostemma* species [27]. Notably, each species manifested distinct and characteristic codon usage patterns.

Synonymous substitutions at the third codon position play a critical role in preserving amino acid diversity, despite not altering the resulting amino acid [8]. GC composition is a common indicator for assessing codon preference, and there is a higher frequency of pyrimidines compared with purines at the third codon position in chloroplast genes [24,28]. Extensive analysis of cp CDSs across 13 Gnetales species revealed an average GC proportion of 36.82% with a staged decline in GC1, GC2, and GC3, indicative of a predominantly AT-rich environment as opposed to GC. As key representatives of the gymnosperms, Gnetales species manifested a terminal nucleotide preference for A or T in their chloroplast genomic codons, with a particular prevalence of T, which is a pattern aligned with eudicots yet distinct from the G or C terminations preferred by monocots [29]. RSCU assessments revealed a lower prevalence of C-terminating or G-terminating codons and a distinct preference for those ending in A or T among the 13 Gnetales species (RSCU > 1). Further, the RFSC and high-frequency codon assessment of these species’ chloroplast genomes validated an AT-ending codon bias, mirroring tendencies also observed in other advanced plant chloroplast genomes, including those in Poaceae [30], Asteraceae [31], Solanaceae [32], and Euphorbiaceae [33]. Earlier studies have illustrated that neutral mutations at the codon’s third locus resulted in the stochastic selection of synonymous codons [28]. Thus, the uneven nucleotide utilization at the third position of cp CDSs in the 13 Gnetales species suggests that CUB was shaped by multiple forces, including but not restricted to mutation pressure and natural selection.

According to the neutral theory of molecular evolution, base mutations and natural selection exert effects on the third codon positions that are typically neutral or nearly so [34]. A dominant influence of natural selection is inferred from a lack of correlation among the base composition trends at the three codon positions [34]. Our current analysis failed to identify a significant correlation between GC content at GC12 and GC3, as indicated by a regression slope approaching null. The results were indicative of a potential natural selection impact on codon selection within Gnetales species. Evaluating the ENC-plot and PR2-plot, it became evident that mutation pressure contributed minimally to CUB within the chloroplast genomes of Gnetales species. Conversely, natural selection appeared as the predominant influence. This observation aligned with findings in prior studies of cp CDSs in *Gynostemma* [27], *Pisum* [35], and *Juglandaceae* [36]. The neutrality analysis further corroborated the primary role of natural selection in defining the CUB of the 13 Gnetales species assessed, consistent with the determinants of CUB in the chloroplast genomes of Leguminosae [37] and *Elaeagnus* [38]. Additionally, COA analyses of relative synonymous codon usage revealed that the first axis explained only a portion of the variation in codon usage. Therefore, we inferred that not only natural selection but also several other factors were likely involved in determining the selective constraints on codon bias in plant chloroplast genomes. Based on our findings in the chloroplast genomes of these Gnetales species, we inferred that natural selection and mutation collectively influenced CUB, with a distinctive emphasis on the former.

In our examination, the SCUO values reported for the majority of the 13 Gnetales species exhibited variability, with all recorded values falling below 0.3. This uniformity indicates a generally low CUB throughout these species, as reduced SCUO values signify weaker CUB [39]. This trend aligns with previously recorded cp CDS analysis for *Oryza* species [26] and Theaceae species [25]. Specifically, the average SCUO values for *Oryza* species and Theaceae species ranged from 0.24 to 0.27 and 0.23 to 0.24, respectively, reflective of low and divergent CUB among these diverse species. Our study further identified high MILC values, spanning from 0.55 to 0.56 among the 13 Gnetales species, suggesting that cp CDSs exhibit relatively high expression levels. Moreover, our results revealed a significant positive correlation between MILC and SCUO values (*p* < 0.01), proposing that CUB potentially influenced gene expression. This association has gained increasing recognition, with studies corroborating a direct relationship between heightened CUB strength and elevated frequencies of gene expression across various plants [40,41]. Additionally, gene length exhibited significant negative correlations with SCUO across all Gnetales species, suggesting that longer gene lengths may be associated with lower CUB, and consequently, reduced gene expression [14]. Similarly, GRAVY, as another factor influenced by natural selection, also demonstrated a significant negative correlation with SCUO across all Gnetales species, reinforcing previous findings that GRAVY plays an important role in shaping the codon usage of genes within a genome [42].

Codon optimization, which can modulate gene expression efficiency, has been suggested as a strategy for transgenic research advancement [8,14]. The chloroplasts of the 13 evaluated Gnetales species demonstrated considerable conservation, with a total of 11 high-frequency codons being commonly shared. Furthermore, each species demonstrated between 2 and 7 species-specific optimal codons, yet no common optimal codon was identified across the 13 representative species. Insights into high-frequency and optimal codons not only assists in refining codon optimization strategies but also broadens our understanding of the connection between codon usage preference and gene expression. Additionally, a comparison of codon usage frequencies between the 13 Gnetales species and 4 model organisms revealed that *S. cerevisiae* and *N. tabacum* would be the preferred heterologous expression hosts for improving successful exogenous gene expression and its subsequent optimization. Consequently, our analysis of the codon usage patterns in the Gnetales chloroplast genomes could have significant implications for the optimization of gene modifications, particularly for those genes essential to growth and development.

## 4. Materials and Methods

### 4.1. Chloroplast Genomes and Data Collection

Chloroplast genomes from 13 different species belonging to the Gnetales order, including Ephedra (*Ephedra equisetina*, *E. foeminea*, *E. intermedia*, *E. sinica*, and *E. monosperma*), *Gnetum* (*Gnetum gnemon*, *G. montanum*, *G. parvifolium*, *G. ula*, *G. hainanense*, *G. pendulum*, and *G. luofuense*), and *Welwitschia* (*Welwitschia mirabilis*) were retrieved from the NCBI GeneBank database (Table S1). To minimize sampling bias and enhance the reliability of findings, the chloroplast coding sequences (cp CDSs) were meticulously filtered according to stringent criteria with a Perl script (https://github.com/xmyangfile/DataAnalysisScripts, accessed on 1 March 2023): (1) each cp CDS must begin with an ATG start codon and end with one of the three termination codons (TGA, TAA, TAG) without any internal stop codons; (2) in each chloroplast CDS, the total number of bases must constitute a multiple of three, to ensure proper translation into amino acids; (3) the cp CDS length must be no less than 300 base pairs [25,26].

### 4.2. Characteristics of Codon Usage Bias Indices

For the evaluation of codon usage patterns, our analysis employed a comprehensive set of metrics: (1) effective number of codons (ENC), indicating codon usage bias; (2) GC content (GC1, GC2, and GC3), along with GC3s, the GC content at the third position of synonymous codons; (3) overall nucleotide composition and each composition at the third codon position (A, T, G, C, A3, T3, C3, and G3); (4) codon adaptation index (CAI), a measure of codon preference in highly expressed genes; (5) the total number of amino acids (L_aa). The CodonW v1.4.2 (http://codonw.sourceforge.net/, accessed on 1 March 2023) and CUSP program (https://www.bioinformatics.nl/cgi-bin/emboss/cusp, accessed on 1 March 2023) were used to calculate these parameters.

### 4.3. Analysis of High-Frequency Codons

The RSCU metric quantitatively assesses codon usage bias by comparing the actual frequency of a specific codon to its expected frequency under the assumption of uniform distribution across all synonymous codons for a given amino acid. A codon’s RSCU value being greater than 1 denotes a pronounced preference for that codon, reflecting its usage at a frequency higher than anticipated. In contrast, an RSCU value less than 1 indicates disfavor, where the codon is utilized less than expected among its synonymous peers. An RSCU value of exactly 1 implies a lack of bias, showcasing an equitable distribution among synonymous codons, thereby suggesting a scenario of stochastic codon selection [43]. The calculation formula of the RSCU is as follows:

(1) $$\mathrm{RSCU}=\frac{X_{\mathrm{ij}}}{\sum_{j}^{n_{i}}X_{\mathrm{ij}}}n_{i}$$

where n_i_ represents the number of synonymous codons encoding the i-th amino acid, and X_ij_ represents the frequency of codon j encoding the i-th amino acid.

The relative frequency of synonymous codons (RFSC) quantifies the occurrence frequency of a specific codon relative to the aggregate count of all synonymous codons for a particular amino acid. The calculation of RFSC is as follows:

(2) $$\mathrm{RFSC}=\frac{X_{\mathrm{ij}}}{\sum_{j}^{N_{i}}X_{\mathrm{ij}}}$$

where X_ij_ refers to the frequency at which the codon j encodes the i-th amino acid.

Codons were classified as high-frequency (HF) based on their RFSC values across the codon spectrum. A codon was deemed HF if its RFSC value exceeded 60% for that specific codon, or if its RFSC was over 50% higher than the mean frequency of all its synonymous counterparts [26].

### 4.4. Analysis of Optimal Codons

ENC can be used to describe the degree of codon usage deviation from random selection. The larger the ENC value, the lower the codon usage bias, and vice versa. After sorting the ENC values of each cp CDS in the 13 different species’ chloroplast genomes, 10% of all filtered cp CDSs with the lowest and highest ENC values were selected and considered as the low- and high-expression genes datasets, respectively. Codons were classified as preferential based on comparisons between low- and high-expression datasets, specifically if ΔRSCU exceeded 0.08, and RSCU values were above 1 for the high-expression group and below 1 for the low-expression group [27].

### 4.5. Synonymous Codon Usage Order (SCUO) Analysis

To measure the relationship between CUB and gene expression, we computed the SCUO for cp CDSs across 13 Gnetales species using the R package “vhcub” [44]. SCUO values, indicative of the degree of synonymous CUB across the entire sequence, span from 0 (minimal bias) to 1 (maximal bias). Lower SCUO values imply a reduced CUB intensity, while increased SCUO values indicate a heightened CUB intensity [39].

### 4.6. Measure Independent of Length and Composition

MILC was a pivotal metric for assessing gene expression levels. MILC reflects the interplay between gene expression levels, gene length, and nucleotide composition. The value of MILC was calculated with the R package “coRdon 1.13.0” (https://github.com/BioinfoHR/coRdon, accessed on 1 March 2023). Lower MILC values indicate reduced gene expression levels, with the inverse implication for higher values [45].

### 4.7. Correspondence Analysis (COA) of Codon Usage

As a multivariate statistical analysis method, COA is widely used to understand the relationships between variables and samples [46]. COA was performed with the RSCU values of individual codons to explore the codon usage patterns of cp CDSs of 13 Gnetales species. Each cp CDS represented a 59-dimensional vector space indicating 59 synonymous codons devoid of ATG, TGG, TAA, TAG, and TGA, where each point represented the RSCU values of the synonymous codon. The maximum fraction of genetic variation could therefore be calculated using the principal trends (Axis 1) of these axes in the 59-dimensional hyperspace, revealing the primary sources of codon usage variation. Furthermore, the correlation indices between Axis 1 and key codon usage metrics, including GC3s, ENC, the total amino acid count in the encoded polypeptide (L_aa), and the codon adaptation index (CAI), were computed using R software (v 4.3.0).

### 4.8. Parity Rule 2 (PR2) Plot Analysis

The PR2-plot was utilized, plotting G3/(G3 + C3) on the x-axis and A3/(A3 + T3) on the y-axis, to examine the composition of the four bases at the third position of codons, which is crucial for understanding the impact of mutation and selection pressure on codon usage patterns [47]. Theoretically, if single mutation pressure was the sole influence on the codons of chloroplast genes, the ratios of A to T and C to G should be equal, leading to a central point on the PR2-plot where both coordinates equal 0.5 (indicating G = C and A = T). Deviations from this point suggest the influence of natural selection and other factors on codon usage.

### 4.9. ENC-Plot Analysis

GC3s denotes the frequency of guanine (G) or cytosine (C) at the third codon position within genes with the exclusion of methionine (Met) and tryptophan (Trp) codons due to their unique, non-synonymous roles in encoding Met and Trp, respectively. The ENC-plot, which maps the ENC against GC3s values, aims to underscore the influence of base composition on codon usage bias and explore the potential impact of other factors on this bias. The benchmark curve for this analysis is derived from the ENC and GC3 values of the 13 species, employing the formula

(3) $$\mathrm{ENC}=2+\mathrm{GC3}+29/[{\mathrm{GC3}}^{2}+{(1\;-\;\mathrm{GC3})}^{2}]$$

ENC values positioned on or near the expected curve indicate that mutation pressure was the primary factor influencing codon usage patterns. Conversely, ENC values located below this curve suggest that natural selection limits codon choice [48].

### 4.10. Neutrality Analysis

Neutrality analysis constitutes a quantitative methodology for unraveling the intricate dynamics between natural selection and mutation pressure influencing codon usage patterns. Employing the average GC content in the first and second codon positions (GC12) for comparison with that at the third position (GC3), this method provides insights into codon bias. A marked correlation between GC12 and GC3, especially when the regression coefficient nears or equals 1, indicates mutation pressure as the primary determinant of codon usage. Conversely, a regression coefficient close to 0, alongside an insignificant correlation, implies that natural selection plays a pivotal role in determining codon preferences [49].

### 4.11. Comparative Analysis of the Frequency of Codon Usage

The ratio of codon usage frequency can be used to indicate the codon usage bias among species. For a deeper analysis of codon usage patterns within the chloroplast genomes of the 13 Gnetales species, we benchmarked against codon usage bias data for 4 model species from the Codon Usage Database (https://www.kazusa.or.jp/codon/, accessed on 1 March 2023), including *Escherichia coli*, *Saccharomyces cerevisiae*, *Nicotiana tabacum*, and *Arabidopsis thaliana*, renowned as prevalent hosts for heterologous expression. A ratio exceeding 0.5 yet below 2 implies a negligible codon bias disparity between two organisms, while values beyond this threshold signify pronounced codon bias distinctions.

### 4.12. Cluster Analysis and Phylogenetic Analysis

To deepen our understanding of the phylogenetic interrelations among Gnetales species, we conducted clustering and phylogenetic analyses using coding sequences from the chloroplast genomes of 13 Gnetales species. After excluding three stop codons (UAA, UAG, UGA) and two singular amino acid codons (UGG and AUG), biased clustering utilized the remaining 59 synonymous codons to compute the squared Euclidean distance based on the RSCU values. We constructed the cluster diagram using the intergroup linkage method in R software (v 4.3.0). All CDSs of the 13 Gnetales species extracted from each chloroplast genome were utilized to construct the maximum likelihood phylogenetic tree with MEGA X software (v 11, https://www.megasoftware.net/ accessed on 1 March 2023) with a bootstrap count of 1000 and other parameters set to the defaults [50]. The optimal nucleotide substitution model of the phylogenetic tree was determined by the ModelTest-NG software (v 0.1.7, https://github.com/ddarriba/modeltest accessed on 1 March 2023) [51].

## 5. Conclusions

Our study provides a systematic analysis of codon usage patterns in the chloroplast genomes of Gnetales species complemented by a comprehensive exploration of factors influencing CUB. The results revealed a strong preference for A/T bases and codons that terminate in A/T within the cp CDSs of all species. Additionally, we identified 20 high-frequency codons and found 2 to 7 optimal codons within the cp CDSs of all species. Our analyses of the ENC-plot, PR2-plot, and neutrality plot revealed that the codon usage patterns in the 13 Gnetales species resulted from a combination of factors, with natural selection playing a predominant role. Correlation analysis indicated natural selection could influence CUB. We identified *S. cerevisiae* and *N. tabacum* as suitable exogenous expression hosts for chloroplast genes in Gnetales species. Both cluster and phylogenetic analyses suggested that even less-preferred codons could play a role in the evolution of organisms. Our discoveries reveal the evolutionary mechanisms of CUB in Gnetales species and may also guide efforts to enhance gene expression efficiency through codon optimization in transgenic studies.

## Figures and Tables

**Table 1:** Optimal codons in chloroplast genomes of 13 Gnetales species.

Species	Optimal Codon Numbers	Optimal Codon
*Ephedra equisetina*	3	‘UUG’, ‘CUU’, ‘UCC’
*Ephedra foeminea*	4	‘AUA’, ‘CUU’, ‘CUA’, ‘ACA’
*Ephedra intermedia*	3	‘AUA’, ‘CUU’, ‘ACA’
*Ephedra monosperma*	3	‘AUA’, ‘CUU’, ‘ACA’
*Ephedra sinica*	4	‘AUA’, ‘CUU’, ‘UCC’, ‘ACA’
*Gnetum gnemon*	5	‘GCA’, ‘CUA’, ‘UCC’, ‘UCG’, ‘ACA’
*Gnetum hainanense*	7	‘GCA’, ‘CGA’, ‘GGC’, ‘CUA’, ‘UCC’, ‘ACC’, ‘ACA’
*Gnetum luofuense*	7	‘GCA’, ‘CGA’, ‘GGC’, ‘CUA’, ‘UCC’, ‘ACC’, ‘ACA’
*Gnetum monotanum*	7	‘GCA’, ‘CGA’, ‘GGG’, ‘CUA’, ‘UCC’, ‘ACC’, ‘ACA’
*Gnetum parvifolium*	6	‘GCA’, ‘CGA’, ‘GGG’, ‘UCC’, ‘ACA’, ‘GUC’
*Gnetum pendulum*	7	‘GCA’, ‘CGA’, ‘GGG’, ‘CUA’, ‘UCC’, ‘ACC’, ‘ACA’
*Gnetum ula*	2	‘CUA’, ‘ACC’
*Welwitschia mirabilis*	6	‘GCC’, ‘AUA’, ‘UUG’, ‘CUA’, ‘UCA’, ‘GUA’

**Table 2:** High-frequency codons in the chloroplast genome of 13 Gnetales species.

Amino Acid	High-Frequency Codons
Leu	UUA
Val	GUU
Ser	UCU
Thr	ACU
Tyr	UAU
Gln	CAA
Lys	AAA
Ala	GCU
Glu	GAA
Arg	AGA, CGU *, CGA *
Gly	GGA
Phe	UUU *
Ile	AUU *
Pro	CCU *
His	CAU
Asn	AAU *
Asp	GAU *
Cys	UGU *

Note: The codon marked with a star on the right indicates high-frequency codons that differed among the chloroplast genomes of the 13 Gnetales species.

**Table 3:** Correlation between SCUO and MILC in the 13 Gnetales species.

Species Name	r	*p*
*Gnetum gnemon*	0.561 **	0.000
*Gnetum montanum*	0.411 *	0.011
*Gnetum parvifolium*	0.455 **	0.004
*Gnetum ula*	0.500 **	0.001
*Gnetum hainanense*	0.492 **	0.002
*Gnetum pendulum*	0.489 **	0.002
*Gnetum luofuense*	0.480 **	0.002
*Welwitschia mirabilis*	0.429 **	0.005
*Ephedra equisetina*	0.071	0.657
*Ephedra foeminea*	0.158	0.324
*Ephedra intermedia*	0.071	0.660
*Ephedra sinica*	0.119	0.460
*Ephedra monosperma*	0.066	0.682

Note: * *p* < 0.05, ** *p* < 0.01.

## Data Availability

All data are contained within the article and its Supplementary Files.

## Supplementary Materials

The following supporting information can be downloaded at: https://www.mdpi.com/article/10.3390/ijms251910622/s1.
