# Supplementary material for: Comparative Analysis of the Codon Usage Pattern in the Chloroplast Genomes of Gnetales Species
Source: Int J Mol Sci. 2024 Oct 2;25(19):10622. doi: 10.3390/ijms251910622 (PMC11477115; doi:10.3390/ijms251910622)
Supplement: Supplementary file 1 [file ijms-25-10622-s001.zip › Table S4.pdf]

Supplementary Table S4 Correlation analysis of axis 1 and codon usage index of chloroplast genomes of 13 Gentale species.

**Species**	**T3s**	**C3s**	**A3s**	**G3s**	**CAI**	**CBI**	**Fop**	**Nc**	**GC3s**	**GC**	**L_aa**
Gnetum gnemon	0.405*	0.073	-0.667**	-0.433**	0.585**	0.412**	0.308	-0.334*	-0.079	0.674**	-0.273
Gnetum montanum	0.436**	0.048	-0.647**	-0.487**	0.555**	0.433**	0.323	-0.366*	-0.135	0.673**	-0.296
Gnetum parvifolium	-0.483**	-0.111	0.705**	0.379*	-0.629**	-0.460**	-0.365*	0.355*	0.024	-0.673**	0.209
Gnetum ula	-0.428**	-0.124	0.619**	0.485**	-0.617**	-0.414**	-0.333*	0.393*	0.125	-0.687**	0.284
Gnetum hainanense	-0.496**	-0.130	0.704**	0.400*	-0.637**	-0.456**	-0.367*	0.336*	0.042	-0.668**	0.261
Gnetum pendulum	0.493**	0.119	-0.710**	-0.394*	0.627**	0.452**	0.362*	-0.333*	-0.039	0.673**	-0.259
Gnetum luofuense	0.391*	0.144	-0.711**	-0.365*	0.438**	0.364*	0.229	-0.280	0.035	0.678**	-0.279
Welwitschia mirabilis	-0.193	-0.124	0.510**	0.587**	-0.305	-0.176	-0.104	0.112	0.203	-0.730**	0.292
Ephedra equisetina	-0.361*	0.131	0.681**	0.403**	-0.355*	-0.194	-0.116	0.434**	0.144	-0.721**	0.321*
Ephedra foeminea	0.364*	-0.146	-0.673**	-0.398**	0.353*	0.193	0.118	-0.389*	-0.145	0.718**	-0.327*
Ephedra intermedia	0.362*	-0.122	-0.676**	-0.417**	0.359*	0.188	0.114	-0.441**	-0.149	0.722**	-0.325*
Ephedra sinica	-0.374*	0.132	0.661**	0.451**	-0.400**	-0.216	-0.140	0.457**	0.178	-0.711**	0.320*
Ephedra monosperma	-0.358*	0.128	0.678**	0.408**	-0.356*	-0.194	-0.119	0.445**	0.144	-0.725**	0.324*

Note: the T, C, A and G content at the third codon position of synonymous codons; codon adaptation index: CAI; codon bias index: CBI; frequency of optimal codons: Fop; the GC content at the third codon position of synonymous codons: GC3s; the GC content at the three position of synonymous codons: GC; total number of amino acids: L_aa)
