# Supplementary material for: Comparative Analysis of the Codon Usage Pattern in the Chloroplast Genomes of Gnetales Species
Source: Int J Mol Sci. 2024 Oct 2;25(19):10622. doi: 10.3390/ijms251910622 (PMC11477115; doi:10.3390/ijms251910622)
Supplement: Supplementary file 1 [file ijms-25-10622-s001.zip › Table S5.pdf]

Supplementary Table S5 Correlation coefficient of SCUO with GC skew, AT skew, AG skew, CT skew, AC skew, and TG skew for cp CDSs in 13 Gentale species.

Species		GC skew	AT skew	AG skew	CT skew	AC skew	TG skew
Gnetum gnemon	SCUO	0.318*	0.318*	0.361*	0.361*	0.368*	0.368*
	P	0.048	0.048	0.024	0.024	0.021	0.021
Gnetum montanum	SCUO	0.276	0.276	0.392*	0.392*	0.395*	0.395*
	P	0.099	0.099	0.016	0.016	0.015	0.015
Gnetum parvifolium	SCUO	0.246	0.246	0.488**	0.488**	0.468**	0.468**
	P	0.136	0.136	0.002	0.002	0.003	0.003
Gnetum ula	SCUO	0.231	0.231	0.421**	0.421**	0.428**	0.428**
	P	0.157	0.157	0.008	0.008	0.007	0.007
Gnetum hainanense	SCUO	0.245	0.245	0.434**	0.434**	0.409*	0.409*
	P	0.138	0.138	0.006	0.006	0.011	0.011
Gnetum pendulum	SCUO	0.238	0.238	0.439**	0.439**	0.413*	0.413*
	P	0.151	0.151	0.006	0.006	0.010	0.010
Gnetum luofuense	SCUO	0.318*	0.318*	0.483**	0.483**	0.423**	0.423**
	P	0.048	0.048	0.002	0.002	0.007	0.007
Welwitschia mirabilis	SCUO	0.290	0.290	0.351*	0.351*	0.339*	0.339*
	P	0.066	0.066	0.024	0.024	0.030	0.030
Ephedra equisetina	SCUO	0.330*	0.330*	0.164	0.164	0.103	0.103
	P	0.035	0.035	0.305	0.305	0.521	0.521
Ephedra foeminea	SCUO	0.350*	0.350*	0.138	0.138	0.084	0.084

	P	0.025	0.025	0.389	0.389	0.602	0.602
Ephedra intermedia	SCUO	0.339*	0.339*	0.171	0.171	0.105	0.105
	P	0.030	0.030	0.286	0.286	0.515	0.515
Ephedra sinica	SCUO	0.326*	0.326*	0.138	0.138	0.072	0.072
	P	0.038	0.038	0.388	0.388	0.657	0.657
Ephedra monosperma	SCUO	0.329*	0.329*	0.165	0.165	0.096	0.096
	P	0.036	0.036	0.303	0.303	0.552	0.552

Note: $^{**}P < 0.01$, $^{*}P < 0.05$
