# Supplementary material for: Comparative Analysis of the Codon Usage Pattern in the Chloroplast Genomes of Gnetales Species
Source: Int J Mol Sci. 2024 Oct 2;25(19):10622. doi: 10.3390/ijms251910622 (PMC11477115; doi:10.3390/ijms251910622)
Supplement: Supplementary file 1 [file ijms-25-10622-s001.zip › Table S6.pdf]

Supplementary Table S6 Correlation coefficients between SCUO and different properties of chloroplast proteins in 13 Gnetale species.

Species		Gene length	GRAVY	Molecular weight	pI (isoelectric point)
Gnetum gnemon	SCUO	-0.572**	-0.559**	-0.244	0.364*
	P	0.000	0.000	0.135	0.023
Gnetum montanum	SCUO	-0.564**	-0.551**	-0.246	0.440**
	P	0.000	0.000	0.141	0.006
Gnetum parvifolium	SCUO	-0.603**	-0.589**	-0.320	0.386*
	P	0.000	0.000	0.050	0.017
Gnetum ula	SCUO	-0.617**	-0.605**	-0.385*	0.399*
	P	0.000	0.000	0.016	0.012
Gnetum hainanense	SCUO	-0.587**	-0.573**	-0.269	0.377*
	P	0.000	0.000	0.102	0.020
Gnetum pendulum	SCUO	-0.589**	-0.576**	-0.273	0.376*
	P	0.000	0.000	0.098	0.020
Gnetum luofuense	SCUO	-0.572**	-0.558**	-0.342*	0.261
	P	0.000	0.000	0.033	0.108
Welwitschia mirabilis	SCUO	-0.587**	-0.572**	-0.223	0.333
	P	0.000	0.000	0.161	0.033
Ephedra equisetina	SCUO	-0.492**	-0.479**	0.013	0.112
	P	0.001	0.002	0.936	0.484
Ephedra foeminea	SCUO	-0.491**	-0.478**	0.030	0.140
	P	0.001	0.002	0.854	0.383
Ephedra intermedia	SCUO	-0.489**	-0.475**	0.007	0.128
	P	0.001	0.002	0.964	0.427
Ephedra sinica	SCUO	-0.486**	-0.473**	0.053	0.098
	P	0.001	0.002	0.742	0.543
Ephedra monosperma	SCUO	-0.494**	-0.481**	0.015	0.116
	P	0.001	0.001	0.924	0.470

Note: *Significant at $P < 0.05$; **Significant at $P < 0.01$
